# MicroRNA Profiling of Salivary Adenoid Cystic Carcinoma: Association of miR-17-92 Upregulation with Poor Outcome

**DOI:** 10.1371/journal.pone.0066778

**Published:** 2013-06-25

**Authors:** Yoshitsugu Mitani, Dianna B. Roberts, Hanadi Fatani, Randal S. Weber, Merrill S. Kies, Scott M. Lippman, Adel K. El-Naggar

**Affiliations:** 1 Department of Pathology, The University of Texas MD Anderson Cancer Center, Houston, Texas, United States of America; 2 Department of Head and Neck Surgery, The University of Texas MD Anderson Cancer Center, Houston, Texas, United States of America; 3 Department of Thoracic/Head and Neck Medical Oncology, The University of Texas MD Anderson Cancer Center, Houston, Texas, United States of America; 4 Moores Cancer Center, University of California San Diego, San Diego, California, United States of America; John Hopkins Medical School, United States of America

## Abstract

**Background:**

Salivary adenoid cystic carcinoma (ACC) is a rare relentlessly progressive malignant tumor. The molecular events associated with ACC tumorigenesis are poorly understood. Variable microRNAs (miRNA) have been correlated with tumorigenesis of several solid tumors but not in ACC. To investigate the association of miRNAs with the development and/or progression of ACC, we performed a comparative analysis of primary ACC specimens and matched normal samples and a pooled salivary gland standard and correlated the results with clinicopathologic factors and validated selected miRNAs in a separate set of 30 tumors.

**Methods:**

MiRNA array platform was used for the identification of target miRNAs and the data was subjected to informatics and statistical interrelations. The results were also collected with the *MYB-NFIB* fusion status and the clinicopathologic features.

**Results:**

Differentially dysregulated miRNAs in ACC were characterized in comparison to normal expression. No significant differences in miRNA expression were found between the *MYB-NFIB* fusion positive and -negative ACCs. Of the highly dysregulated miRNA in ACC, overexpression of the miR-17 and miR-20a were significantly associated with poor outcome in the screening and validation sets.

**Conclusion:**

Our study indicates that the upregulation of miR-17-92 may play a role in the biology of ACC and could be potentially targeted in future therapeutic studies.

## Introduction

Adenoid cystic carcinoma, an uncommon salivary gland malignancy, is characterized by histopathologic and cellular heterogeneity and a relentless progressive clinical course [1], [2]. The primary treatment of ACC is complete surgical excision with and without post-operative radiotherapy [3]. Patients with locally advanced primary, recurrent, and metastatic ACC have been treated experimentally with chemotherapy and targeted agents with minimal success [4], [5]. Several genomic investigations exclusive of miRNA analysis have been carried out in ACC to identify biological markers of therapeutic potential [6]–[9]. These efforts, however, have been largely unrewarding and additional investigations of new targets are needed.

MiRNAs, a new class of highly conserved, short (19-22-nucleotides) non-coding RNA molecules, are products of a highly coordinated processing of a long RNA sequence template by specific RNAase III endonucleases [10]. Most miRNAs' regulatory functions are achieved through binding to the 3′ untranslated sequence of the RNA target (3′-UTR) transcript. Complete complementarity of miRNA to their messenger RNA targets results in complete transcriptional repression, while imperfect matching, the most common occurrence lead to partial transcriptional dysregulation. Imperfect or partial base-pairing with target mRNAs, however, allows the miRNA to bind to a large number of coding genes. Moreover, multiple miRNAs can be produced from a single pre-miRNA transcript and these may act independently or in concert on a wide range of genes in both normal and tumorigenic status [11]–[15].

Except for a study of miRNA expression in pleomorphic adenoma, a common benign salivary gland tumor, little is known about the role of these molecules in malignant salivary tumors including ACC [16]. Recently, a t(6; 9) leading to a fusion between the *MYB* and *NFIB* genes and the *MYB* gene overexpression was reported in a large subset of ACCs [17], [18]. Interestingly, the upregulation of MYB in ACC has been suggested due to the disruption of the 3′ UTR (miRNA binding sites) by the translocation with the *NFIB* gene [19]–[21]. Furthermore, evidence for a regulatory effect of the *MYB* gene on other miRNAs has been shown [22]. Collectively, these findings suggest a role for certain miRNAs in ACC tumorigenesis.

We hypothesize that certain miRNAs play a role in the regulation of cellular pathways in the ACC tumorigenesis and this may be influenced by the fusion gene status. To test our hypothesis we performed miRNA analysis on normal salivary tissues and *MYB-NFIB* fusion positive and negative ACCs to determine differentially altered candidates of potential biological significance.

## Materials and Methods

### Ethics Statement

This study was approved by the MD Anderson Cancer Center Institutional Review Board (IRB protocol # Lab07-0382). Written informed consent was provided by all patients in this study to perform the subsequent analyses.

### Tissue samples and RNA extractions

For the screening of miRNA expression profiling, fresh frozen tissue specimens from 30 primary ACCs and 4 matched normal salivary samples were collected initially. For the validation of identified miRNAs, 30 further ACC tumor samples were used. All tissue samples were accessioned at the head and neck section, MD Anderson Cancer Center, from 1989 to 2010, and formed the materials for this study. The clinicopathological features were described in Table 1. All tissues were harvested immediately in fresh state and placed in liquid nitrogen and stored at −80°C until used. Total RNA was extracted with the Trizol reagent (Invitrogen, Carlsbad, CA, USA), and then cleaned by RNeasy mini cleanup kit (Qiagen, Hilden, Germany) according to the manufacturer's instructions. The quality of the total RNA was verified by an Agilent 2100 Bioanalyzer profile. All the samples had an RNA integrity number greater than 7.0.

**Table 1:** Demographic and clinicopathologic characteristics of the initial screening (*n* = 30) and the validation sets (*n* = 30) of salivary adenoid cystic carcinoma.

Characteristic	Screening set (N, %)	Validation set (N, %)
Gender		
Male	20 (67)	18 (60)
Female	10 (33)	12 (40)
Age (years)		
<60	20 (67)	22 (73)
≥60	10 (33)	8 (27)
Tumor site		
Major	6 (20)	6 (20)
Minor	24 (80)	24 (80)
Tumor size		22 (73)
<4cm	15 (50)	8 (27)
≥4cm	15 (50)	
Histologic type		
T/C	16 (53)	15 (50)
Any solid	14 (47)	15 (50)
PNI^a^		
No	1 (3)	3 (10)
Yes	28 (93)	19 (63)
Not stated	1 (3)	8 (27)
Stage		
I or II	2 (7)	10 (33)
III or IV	23 (77)	12 (40)
NA	5 (17)	8 (27)
Recurrence		
No	9 (30)	12 (40)
Yes	21 (70)	18 (60)
Distant metastasis		
No	16 (53)	15 (50)
Yes	14 (47)	15 (50)
*MYB-NFIB* fusion^b^		
No	10 (33)	11 (37)
Yes	20 (67)	19 (63)

N; Number.^ a^PNI; Perineural invasion, T/C; Tubular/Cribriform.^ b^ *MYB-NFIB* fusion was identified by FISH (refs. 17 and 21).

### MiRNA array profiling

One µg total RNA from tumor and normal tissue samples and a pooled normal salivary gland standard (Clontech Laboratories) was labelled with Hy3™ and Hy5™ fluorescent label, respectively, using the miRCURY™ LNA Array power labelling kit (Exiqon, Vedbaek, Denmark) following the procedure described by the manufacturer. The Hy3™-labeled samples and a Hy5™-labeled reference RNA sample were mixed pair-wise and hybridized to the miRCURY™ LNA *Array version 5^th^ Generation* (Exiqon), which contains capture probes targeting all miRNAs for human, mouse or rat registered in the miRBASE version 15.0 at the Sanger Institute. The hybridization was performed according to the miRCURY™ LNA array manual using a Tecan HS4800 hybridization station (Tecan, Männedorf, Switzerland). After hybridization, the microarray slides were scanned and stored in an ozone free environment (ozone level below 2.0 ppb) in order to prevent potential bleaching of the fluorescent dyes. The miRCURY™ LNA array microarray slides were scanned using the Agilent G2565BA Microarray Scanner System (Agilent Technologies, Santa Clara, CA, USA) and the image analysis was carried out using the ImaGene 8.0 software (BioDiscovery, El Segundo, CA, USA). The quantified signals were background corrected (Normexp with offset value −10) [23] and normalized using the global Lowess (Locally Weighted Scatterplot Smoothing) regression algorithm.

### MiRNA array data and statistical analysis

The ratios of median values for expression of each miRNA tumor/normal tissues were determined and compared using Mann-Whitney U tests. A cut off of values <0.05 (under-expression) or >2 (over-expression), coupled with a *p*-values of <0.05 by Mann-Whitney U tests, were considered significant. To investigate the effect of the *MYB-NFIB* fusion, the miRNA expression in fusion positive and negative tumors were compared. Fusion status was decided by using fluorescence in-situ hybridization (FISH) on touch preparation of fresh tissues as previously described [17], [21]. For association of miRNAs with clinicopathological parameter and histological patterns of ACC, the median ratio values of each miRNA were compared by Mann-Whitney U test. The significant analysis of microarrays (SAM) algorithm were performed [24] and all miRNAs listed in the false discovery rate (FDR<0.05%) were calculated. For visualization, the linkage clustering with centrered Peason correlation was performed by Multiexperiment Viewer (MeV, http://www.tm4.org/mev.html) tool.

For assessing the correlation of individual miRNAs with survival outcomes, the median ratio values of each miRNA from tumors of patients who had died and who were still living at last contact were compared by Mann-Whitney U test. The direction of the difference in median ratios of those miRNAs that showed statistical significance was determined from the ratios of the median values from tumors of dead/living patients. If the median ratio value of the miRNAs of patients who had died was higher than that from living patients, then the highest quartile of values of all 30 tumor patients were tested against the lower quartile values by the log rank test for survival plots. If the median ratio value of miRNA from patients who had died was lower than that from patients who are still living, then the lowest quartile of values from all 30 tumor patients were similarly tested for correlation with lower survival curves. Those miRNAs that showed statistically significant correlations (p<0.05) in both Mann-Whitney U tests and log rank tests were considered to be significantly correlated with unfavorable survival outcomes. To help determine the magnitude of the effect, Risk Ratios were calculated by Cox regression analysis.

### Quantitative RT-PCR of miRNA

For validation, we selected highly dysregulated miRNAs to be tested by quantitative RT-PCR in a separate set of 30 tumors. The miRUCURY LNA^TM^ Universal RT miRNA PCR assays (Exiqon) for hsa-miR-455-3p, miR-455-5p, miR-375, miR-142-3p, miR-17, and miR-20a were used for their quantification. Five ng of total RNA was used for reverse transcription by Universal cDNA synthesis kit (Exiqon) according to the manufacturer's instructions. Quantitative RT-PCR reactions were run using the 7900HT Fast Real-Time PCR System (Applied Biosystems) with SYBR® Green Master Mix Universal RT (Exiqon) in the Quantitative Genomics Core Laboratory (The University of Texas Health Science Center at Houston). Hsa-miR-99b* was selected as a normalisation primer by NormFinder software [25]. The mean Ct was determined from duplicate reactions. The expression of each miRNAs were determined by the ΔCT method (Average CT-target miRNA – Average CT-miR-99*).

## Results

### MiRNA expression profiles in ACC tumor

**Figure 1:**
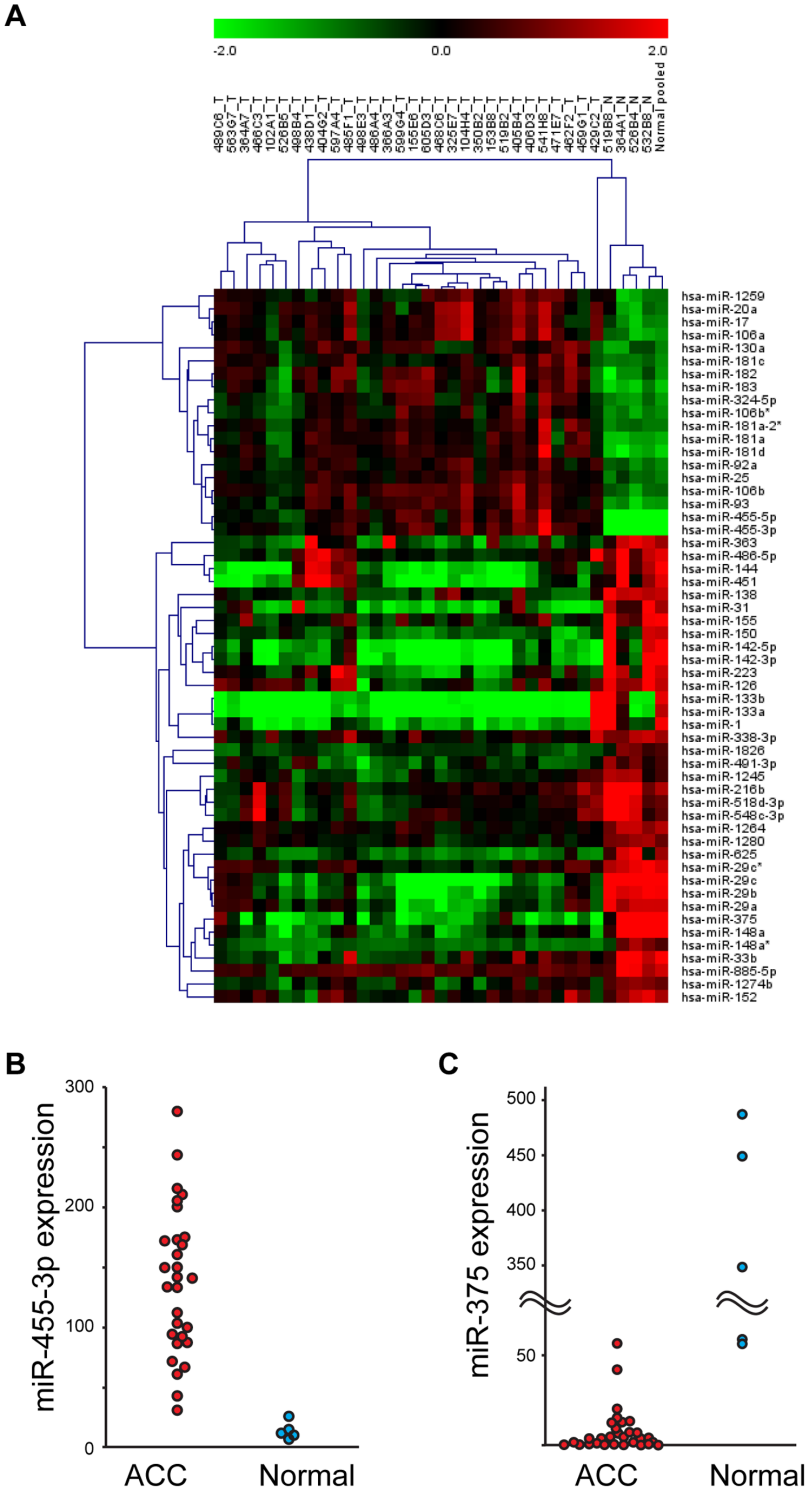
MiRNA expression differences in salivary ACCs and normals. (A) A heat map of the differential miRNA expression in normal salivary tissues and control versus tumor samples. Note the clean distinction between normal tumors. Quantitative RT-PCR validation of miRNA expression, miR-455-3p (B) and miR-375 (C).

Table 1 presents the clinicopathologic characteristics of the 30 ACCs from the screening set and figure 1A, the differentially expressed miRNAs and the heat map diagram of the two-way hierarchical clustering of tumor and normal specimens. The results showed 55 miRNAs to be significantly different between ACCs and normal specimens by both Mann-Whitney U test and SAM algorithm. Nineteen miRNAs were up-regulated (Table 2) and 36 were down-regulated (Table 3) in tumor in comparison to normal and standard. Eight of the 19 up-regulated miRNAs in ACCs represented the miR17-92 cluster and its paralogs, miR106 b-25 and miR106a-363. All highly dysregulated miRNAs were selected for validation by quantitative RT-PCR. Figure 1B and 1C presents the expression of miR-455-3p (up-regulated) and miR-375 (down-regulated) in both normal and tumor specimens; miR455-3p shows high levels of expression in tumor specimens in contrast to normal tissues (*p*<0.001), paradoxically, miR-375 (*p*<0.001) was markedly down regulated in tumors in contrast to normal tissues (Mann-Whitney U test).

**Table 2:** Upregulated miRNAs in salivary adenoid cystic carcinoma in comparison to normal salivary gland.

miRNAs	*p* a	T/N ratio^b^	Chr^c^	Host Gene^d^
hsa-miR-455-3p	0.001	10.75	9q32	*COL27A1*
hsa-miR-455-5p	0.001	7.11	9q32	*COL27A1*
hsa-miR-181d	0.001	3.63	13p.13	*intergenic*
hsa-miR-183	0.001	3.53	7q32.2	*intergenic*
hsa-miR-181a	0.001	3.04	9q33.3	*NR6A1*
^e^hsa-miR-93	0.001	2.96	7q22.1	*MCM7*
hsa-miR-182	0.001	2.86	7q32.2	*intergenic*
^e^hsa-miR-106a	0.001	2.80	Xq26.2	*intergenic*
^e^hsa-miR-17	0.001	2.66	13q31.3	*MIR17HG*
hsa-miR-130a	0.001	2.56	11q12.1	*AP000662.4*
^e^hsa-miR-20a	0.001	2.43	13q31.3	*MIR17HG*
hsa-miR-324-5p	0.001	2.35	17p13.1	*ACADVL*
^e^hsa-miR-106b	0.001	2.29	7q22.1	*MCM7*
hsa-miR-181a-2*	0.001	2.21	9q33.3	*MIR181A2HG, NR6A1*
hsa-miR-181c	0.001	2.16	19p13.13	*intergenic*
hsa-miR-1259	0.004	2.11	NA	NA
^e^hsa-miR-106b*	0.001	2.09	7q22.1	*MCM7*
^e^hsa-miR-25	0.001	2.07	7q22.1	*MCM7*
^e^hsa-miR-92a	0.001	2.01	13q31.3	*MIR17HG*

NA; no information. miRNA(*); miRNA star strand.

a Mann-Whitney U test. ^b^Tumor/Normal median ratio >2 classified as significant. ^c^Chromosomal location. ^d^These information obtained from miRBase database (http://www.mirbase.org). ^e^The miR-17-92 cluster and its paralogs.

**Table 3:** Downregulated miRNAs in salivary adenoid cystic carcinoma in comparison to normal salivary gland.

miRNAs	*p* a	T/N ratio^b^	Chr^c^	Host Gene^d^
hsa-miR-375	0.001	0.042	2q35	*MIR375*
hsa-miR-142-3p	0.002	0.084	17q22	*intergenic*
hsa-miR-142-5p	0.002	0.096	17q22	*intergenic*
hsa-miR-148a	0.001	0.098	7p15.2	*intergenic*
hsa-miR-29c	0.001	0.102	1q32.2	*intergenic*
hsa-miR-133b	0.003	0.112	6p12.2	*RP11-771D21*
hsa-miR-144	0.002	0.138	17q11.2	*intergenic*
hsa-miR-133a	0.004	0.138	18q11.2	*MIB1*
hsa-miR-29b	0.001	0.158	7q32.3	*AC016831.7*
hsa-miR-31	0.001	0.161	9p21.3	*RP11-354P17.9*
hsa-miR-451	0.007	0.174	17q11.2	*intergenic*
hsa-miR-216b	0.001	0.186	2p16.1	*AC011306.2*
hsa-miR-33b	0.001	0.193	17p11.2	*SREBF1*
hsa-miR-150	0.008	0.212	19q13.33	*intergenic*
^e^hsa-miR-363	0.003	0.214	Xq26.2	*intergenic*
hsa-miR-223	0.008	0.221	Xq12	*AL034397.1*
hsa-miR-155	0.001	0.234	21q21.3	*MIR155HG*
hsa-miR-625	0.001	0.235	14q23.3	*FUT8*
hsa-miR-29a	0.001	0.254	7q32.3	*MIR29A, AC016831.7*
hsa-miR-138	0.001	0.259	16q13	*intergenic*
hsa-miR-518d-3p	0.002	0.303	19q13.42	*intergenic*
hsa-miR-1	0.002	0.305	18q11.2	*MIB1*
hsa-miR-548c-3p	0.005	0.313	12q14.2	*RASSF3*
hsa-miR-486-5p	0.005	0.318	8p11.21	*ANK1*
hsa-miR-148a*	0.001	0.319	7p15.2	*intergenic*
hsa-miR-29c*	0.001	0.322	1q32.2	*intergenic*
hsa-miR-1245	0.001	0.335	2q32.2	*COL3A1*
hsa-miR-126	0.001	0.372	9q34.3	*EGFL7*
hsa-miR-1264	0.001	0.395	Xq23	*HTR2C*
hsa-miR-1274b	0.001	0.449	NA	NA
hsa-miR-338-3p	0.002	0.451	17q25.3	*AATK*
hsa-miR-491-3p	0.001	0.466	9p21.3	*KIAA1797*
hsa-miR-152	0.002	0.470	17q21.32	*COPZ2*
hsa-miR-1826	0.001	0.480	NA	NA
hsa-miR-1280	0.001	0.486	3q21.3	*EEFSEC*
hsa-miR-885-5p	0.005	0.498	3p25.3	*ATP2B2*

NA; no information. miRNA(*); miRNA star strand.

a Mann-Whitney U test,^ b^Tumor/Normal median ratio <0.5 classified as significant. **^c^**chromosomal location. ^d^These information obtained from miRBase database (http://www.mirbase.org). ^e^The miR-17-92 cluster and its paralogs.

### Correlation of miRNA levels and clinicopathologic parameters

Correlation with clinicopathologic factors revealed 108 dysregulated miRNAs to be correlated significantly with tumor size (T), 18 with tumor stage (T), 13 with lymph node metastasis (N), and 39 with tumor recurrence (*p*<0.05, Mann-Whitney U test). Only two miRNAs, let-7a and miR-150, were significantly correlated with T or N and Stage (Table 4); over-expression of miR-let-7a was also correlated with tumor recurrence (Table 4).

**Table 4:** Correlation of miRNAs and Tumor size, Lymph node status, Stage, and Recurrence in patients with adenoid cystic carcinoma.

miRNAs	T	N	Stage	Recurrence
hsa-let-7a	0.012	ns	0.027	0.04
hsa-miR-150	ns	0.019	0.013	ns

*P-*value by Mann-Whitney U tests. T; Tumor size, N; Lymph node status, ns; Not significant.

We also noted that the expression of 133 miRNAs to be significantly associated with tumor that contained solid component. Fifty-five of these were significant in 2-tailed Fisher exact tests and thirty by SAM algorithm (Table 5), including miR-20a and miR-17 (T/N ratio; miR-20a, 1.34, miR-17a, 1.41).

**Table 5:** Correlations of miRNAs with histologic pattern of adenoid cystic carcinoma.

miRNAs	Mann-Whitney U *p* a	Fisher Exact *p* a	S/N ratio^b^
hsa-miR-205	0.009	0.002	0.15
hsa-miR-381	0.005	0.039	0.28
hsa-miR-143	0.013	0.002	0.33
hsa-miR-145	0.009	0.002	0.34
hsa-miR-376c	0.001	0.039	0.35
hsa-miR-145*	0.009	0.002	0.35
hsa-miR-34c-5p	0.002	0.039	0.37
hsa-miR-376a*	0.001	0.039	0.38
hsa-miR-136	0.001	0.039	0.39
hsa-miR-143*	0.007	0.002	0.39
hsa-miR-127-3p	0.001	0.039	0.41
hsa-miR-409-3p	0.001	0.039	0.41
hsa-miR-654-3p	0.001	0.039	0.41
hsa-miR-154	0.001	0.039	0.42
hsa-miR-410	0.001	0.039	0.42
hsa-miR-411	0.003	0.039	0.43
hsa-miR-379	0.001	0.039	0.45
hsa-miR-382	0.001	0.039	0.45
hsa-miR-136*	0.001	0.039	0.46
hsa-miR-377	0.002	0.039	0.46
hsa-miR-329	0.001	0.039	0.50
hsa-miR-432	0.001	0.039	0.52
hsa-miR-22	0.005	0.039	0.53
hsa-miR-495	0.001	0.039	0.56
hsa-miR-127-5p	0.001	0.039	0.59
hsa-miR-376b	0.001	0.039	0.59
hsa-miR-20a	0.013	0.002	1.34
hsa-miR-17	0.010	0.002	1.41
hsa-miR-9*	0.003	0.002	4.49
hsa-miR-9	0.002	0.002	4.59

miRNA(*); miRNA star strand.

a *P*-values were calculated using the Mann-Whitney U test and 2-tailed Fisher exact test for solid and non-solid types. ^b^S/N ratio; Solid/non-solid median ratio.

### Correlation of MiRNA levels and survival

**Figure 2:**
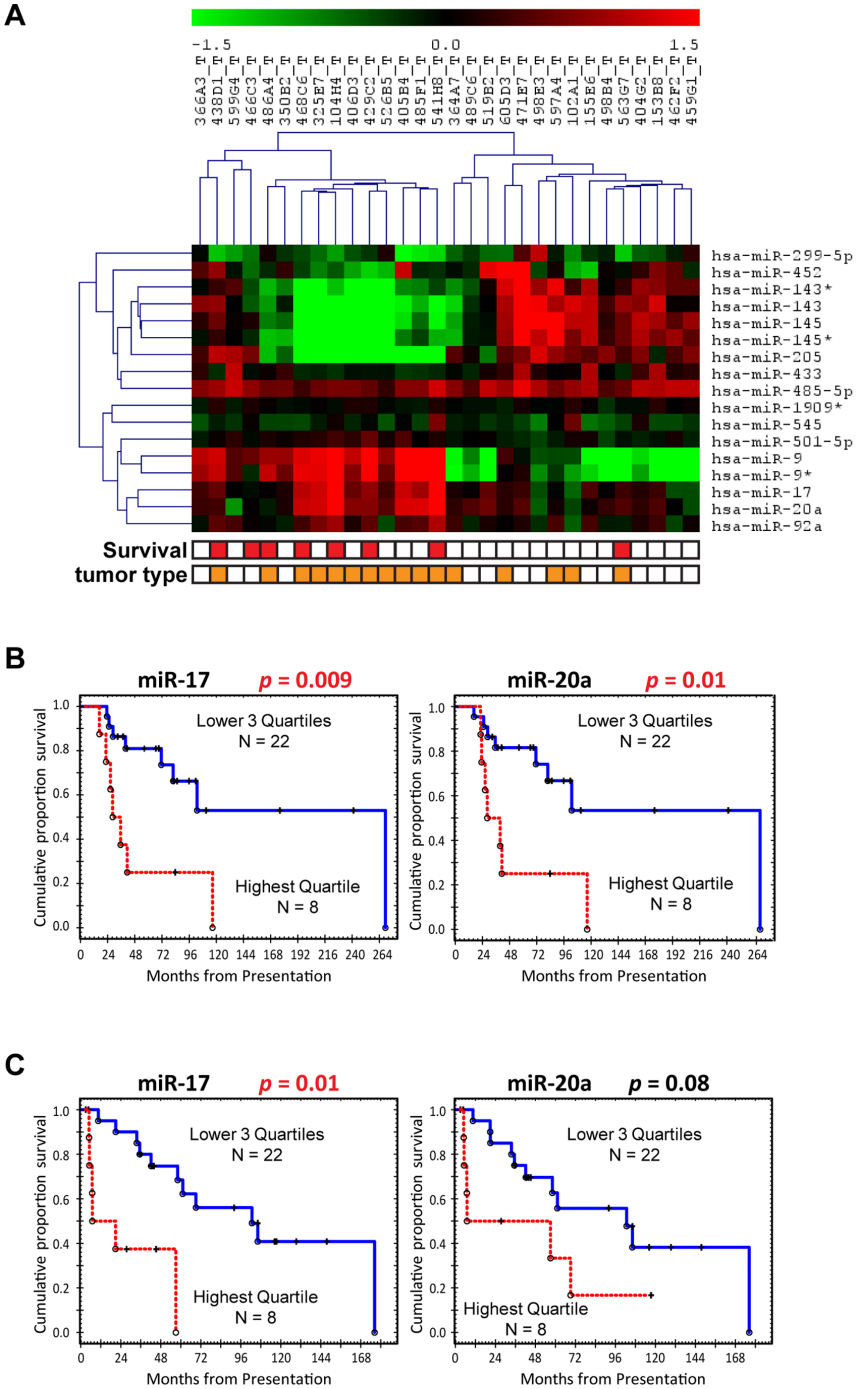
Identification of dysregulated miRNAs for overall survival in ACCs. (A) Hierarchical clustering of the dysragulated miRNAs related to the patients' survival (Table 6) in ACCs. The samples marked by red color in the survival section indicate that the patient died within 3 years. The samples marked by orange color in the tumor type indicate the solid ACC. (B) Kaplan-Meier analysis of quantitative RT-PCR using the screening set of ACCs. High expression of miR-17 and miR-20a is associated with the poor survival significantly (*p* = 0.009 and *p* = 0.01, respectively). The cercle and cross marks indicate the date of death and last contact, respectively. (C) Kaplan-Meier analysis of quantitative RT-PCR using the validation set of ACCs. Further validation confirmed that high expression of miR-17 is correlated significantly with the poor survival (*p* = 0.01).

Figure 2A presents the unsupervised hierarchical clustering of miRNAs expression and patients' survival and Table 6 presents the Log Rank test and Cox proportional hazards regression analysis. Seventeen of the highly dysregulated miRNAs were significantly correlated with poor survival outcomes (Hazard ratios of 2.9 and 4.4). The most significantly correlated miRNAs with survival were the miR-17-92 cluster in a screening set by quantitative RT-PCR (miR-17, and -20a; Figure 2B, Log-rank test, *p* = 0.009 and *p* = 0.01 respectively).

**Table 6:** Correlation between dysregulated miRNAs and clinical outcome in adenoid cystic carcinoma patients.

miRNAs	Log rank *p* a	Expression	HR (95% Cl)^b^	Cox regression *p* a
hsa-miR-433	0.025	Low	4.43 (1.34–14.7)	0.015
hsa-miR-143	0.008	Low	4.37 (1.51–12.7)	0.007
hsa-miR-145	0.008	Low	4.37 (1.51–12.7)	0.007
hsa-miR-501-5p	0.008	High	4.37 (1.51–12.7)	0.007
hsa-miR-483-5p	0.035	Low	3.93 (1.19–12.9)	0.024
hsa-miR-545	0.025	High	3.92 (1.31–11.8)	0.015
hsa-miR-9	0.012	High	3.87 (1.34–11.2)	0.012
hsa-miR-9*	0.012	High	3.87 (1.34–11.2)	0.012
hsa-miR-143*	0.014	Low	3.70 (1.28–10.7)	0.016
hsa-miR-145*	0.014	Low	3.70 (1.28–10.7)	0.016
hsa-miR-17	0.014	High	3.65 (1.27–10.5)	0.016
hsa-miR-20a	0.014	High	3.65 (1.27–10.5)	0.016
hsa-miR-1909*	0.035	High	3.56 (1.17–10.8)	0.025
hsa-miR-299-5p	0.036	Low	3.47 (1.15–10.5)	0.027
hsa-miR-92a	0.040	High	3.21 (1.11–9.34)	0.032
hsa-miR-205	0.046	Low	3.08 (1.06–8.99)	0.039
hsa-miR-452	0.039	Low	2.88 (1.00–8.32)	0.049

miRNA(*); miRNA star strand.

a *P*-values were calculated by Log rank test and Cox regression analysis. ^b^HR; Hazard ratio, CI; confidence interval.

### MiRNA and MYB-NFIB fusion in ACCs

To test whether the fusion status affects miRNA expression, comparative analysis of fusion positive and fusion negative ACC specimens was performed on the 30 screening set; 20 tumors were fusion positive and 10 were fusion negative. The results showed no significant difference in miRNA expression between fusion positive and negative ACCs by SAM. We also found no association between the expression of miR-15a, miR-16 and miR-150 reported to regulate the *MYB* gene through their binding to its 3′UTR [18].

### Validation of miR-17-92 cluster as prognostic miRNAs

We selected members of the miR-17-92 cluster, miR-17 and -20a, for validation in a separate set of 30 tumors by quantitative RT-PCR analysis. In the validation set, no significant correlation was found between the expression levels of both miR-17- and -20a and tumors with solid component. This may be explained by the variable and subjective nature of assessing this component in tumors. Kaplan-Meier analysis shows that there was a significant correlation between miR-17 (Figure 2C, Log-rank test, *p* = 0.01) and strong association between miR-20a expression and poor outcome (Figure 2C, Log-rank test, *p* = 0.08). In combined analysis sets of the 60 ACCs (screening and validation sets), significant statistical association of high expression of miR-17 and miR-20a and poor survival was found (Log-rank test, *p*<0.01). As to the fusion status of the 30 validation set, 19 were fusion positive and 11 were fusion negative, there was no significant difference in the miRNA expression between fusion positive and negative tumors.

## Discussion

As the first miRNA study of ACC, we identified differentially expressed miRNAs that distinctly separated normal salivary tissue and standard from ACC tumors. The most significantly over-expressed miRNAs in tumors were the miR-17-92 cluster and its paralogs including miR-455-3p, -455-5p, -181 and miR-183. In this study, upregulation of miR-17 and miR-20a, members of miR-17-92 cluster genes, was found to be significantly associated with the poor outcome by two different statistical methods. [26]–[37]. Interestingly, evidence for an association between these miRNAs and salivary gland development has been reported [26] suggesting a tissue/tumor context association. This is further supported by the finding that several of the highly dysregulated miRNAs in ACC were found to be involved in head and neck squamous carcinoma biology [38]. The miR-17-92 cluster are encoded by a polycistronic gene on chromosome 13q31 region and are conserved in all vertebrates and play a fundamental regulatory function in the development and progression of several tumor types [26]–[28], [34], [39]–[41]. Surprisingly, two of the highly upregulated miRNAs, (miR-455-3p and miR-455-5p) in our study are of an unknown function. Although the oncogenic and/or functional role of these miRNAs in ACC tumorgenesis and progression are currently unclear, future investigations are needed to determine their biological role in ACC.

In this study, marked down-regulation of miR-375, -142-3p, 142-5p, -148, -155, -33b, and miR-29 family members in ACC was noted. Interestingly, low expression of these miRNAs was also found to be associated with aggressive behavior in several solid neoplastic entities [29], [31], [42]–[50]. Although no correlation between the down regulation of these miRNAs and adverse features of ACC was found, we contend that larger cohorts with long term follow-up is needed to determine their biological role.

Our results show several miRNAs to be correlated with the solid component and poor outcome in patients with ACC. However, our validation analysis in a separate cohort confirmed only the association of the selected miRNAs upregulation with the outcome. The data collectively suggest that the presence of the solid component and poor outcome are not mutually exclusive and these miRNAs play a role in the biological progression of ACC. The data also highlights the difficulties in assessing the extent and the contribution of the solid feature in this entity. Of particular interest, however, is the finding of significant correlation between low miR-205 expression and poor survival in patients with ACC. This miRNA has been reported to be exclusively expressed in the cytoplasm of myoepithelial cells in normal and hyperplastic breast tissues and its loss or down-regulation was significantly associated with progression to ductal carcinoma [51], [52]. We, therefore, contend that the loss of this miRNA in ACC could be attributed to the loss of myoepithelial cells in the solid form.

In this study, no significant correlation between miRNA expression and the fusion status of ACCs was found. We also found no dysregulation of miR-16, 16 and 150 in this cohort. These findings are at variance with previous studies implicating these miRNAs in the regulation of the *MYB* gene [18], [53], [54]. Since one of the major targets of this miRNA is the *MYB* gene, our results suggest that mechanisms other than 3′ UTR deletion are involved in its regulation in ACC [54]. Recently, copy number abnormalities and genomic rearrangements have been shown to influence miRNA in different tumor types; similar integrative analysis may also be needed in ACC [10], [55]. In conclusion, our data shows that upregulation of the miR-17-92 cluster was associated with the aggressive behavior of ACC tumors.
